# 分子印迹聚合物在极性农药残留检测中的应用进展

**DOI:** 10.3724/SP.J.1123.2021.03005

**Published:** 2021-09-08

**Authors:** Ting LI, Mengmeng CHANG, Xianzhe SHI, Guowang XU

**Affiliations:** 1.中国科学院大连化学物理研究所, 中国科学院分离分析化学重点实验室, 辽宁 大连 116023; 1. CAS Key Laboratory of Separation Science for Analytical Chemistry, Dalian Institute of Chemical Physics, Chinese Academy of Sciences, Dalian 116023, China; 2.大连理工大学张大煜学院, 辽宁 大连 116024; 2. Zhang Dayu School of Chemistry, Dalian University of Technology, Dalian 116024, China; 3.中国科学院大学, 北京 100049; 3. University of Chinese Academy of Sciences, Beijing 100049, China

**Keywords:** 极性农药残留, 分子印迹聚合物, 样品前处理, 复杂基质, 综述, polar pesticide residues, molecularly imprinted polymers (MIPs), sample pretreatment, complex matrix, review

## Abstract

极性农药包括杀菌剂、除草剂、杀虫剂等,种类丰富,成本低廉,在农业中应用广泛,其滥用易导致水资源和土壤等环境污染,人类通过间接接触动植物源性食品和环境中的极性农药残留也增加了农药暴露风险。极性农药的物理化学性质差异大,通常痕量存在于食品和环境样品等复杂基质中,这对其准确检测分析带来了挑战。分子印迹聚合物(MIPs)作为一种人工制备的选择性吸附剂,具有与模板分子在空间结构、大小尺寸和功能基团上互补的特定识别位点,且易于制备,成本低,稳定性好,重复利用率高,已被广泛用于极性农药残留的样品前处理和分析检测中。MIPs可以作为固相萃取(SPE)、固相微萃取(SPME)、磁性固相萃取(MSPE)、搅拌棒固相萃取(SBSE)等前处理方法的吸附剂,还可用于制备光、电、化学传感器,作为质谱检测的离子源基底和拉曼光谱的增强基底。目前针对极性农药残留的检测,已有许多研究报道了多种分子印迹材料用于高效分离分析各种复杂基质中的极性农药残留,但未见此方面的综述报道。该文首先介绍了MIPs的印迹策略、聚合策略,并针对传统MIPs制备和应用中存在的问题,简要概括了一些新型的分子印迹策略和制备技术;然后从极性农药残留分析的角度出发,总结归纳了分子印迹材料近年来特别是近5年来在各种极性农药残留(包括新烟碱类、有机磷类、三嗪类、唑类、脲类等)检测中的应用,并针对现存问题展望了其未来的发展方向和趋势。

## Abstract (en)

Polar pesticides can be primarily classified as fungicides, herbicides, and insecticides; their rich variety and low cost have led to their extensive utilization in agriculture. However, the overuse of polar pesticides can lead to environmental contamination, such as water or soil pollution, which can also increase the risk of pesticide exposure among human life directly, or indirectly through contact with animal and plant-derived food. There are considerable differences in the physical and chemical properties of polar pesticides, as well as their trace amounts in complex food and environmental samples, posing immense challenges to their accurate detection. As a kind of artificially prepared selective adsorbent, molecularly imprinted polymers (MIPs) possess specific recognition sites complementary to template molecules in terms of the spatial structure, size, and chemical functional groups. With many advantages such as easy preparation, low cost, as well as good chemical and mechanical stability, MIPs have been widely applied in sample pretreatment and the analysis of polar pesticide residues. MIPs are typically used as adsorption materials in solid phase extraction (SPE) methods, including magnetic solid phase extraction (MSPE), dispersed solid phase extraction (DSPE), and stir bar sorptive extraction (SBSE). To rapidly detect polar pesticide residues with high sensitivity, MIPs are also used in the preparation of fluorescent sensors and electrochemical sensors. Furthermore, MIPs can be employed as the substrate in surface-enhanced Raman spectroscopy and as the substrate for the ion source in mass spectrometry for polar pesticide residue analysis. Thus far, various molecularly imprinted materials have been reported for the efficient separation and analysis of polar pesticide residues in various complex matrices. However, there is no review that summarizes the recent advances in MIPs for the determination of polar pesticides. This review introduces imprinting strategies and polymerization methods for MIPs, and briefly summarizes some new molecular imprinting strategies and preparation technologies. The application of MIPs in recent years (particularly the last five years) to the detection of polar pesticide residues including neonicotinoids, organophosphorus, triazines, azoles, and urea is then systematically summarized. Finally, the future development direction and trends for MIPs are proposed considering existing challenges, with the aim of providing reference to guide future research on MIPs in the field of polar pesticide residue detection.

极性农药定义为正辛醇水分配系数log *P*<4.5的农药^[1,2]^,包含杀菌剂、除草剂、杀虫剂等类别,并且不断发展出新的化学类型。极性农药在农业、家庭(如花园和宠物杀虫剂)、公共卫生和工业中应用广泛,通过食物链的传递或者通过环境摄入进入人体,对人类的健康造成潜在威胁^[3,4]^。世界上很多国家和地区越来越意识到极性农药残留带来的风险,规定了极性农药的最大残留限量。因此准确检测食品和环境中的极性农药显得尤为迫切。目前常用气相色谱、液相色谱结合质谱进行农药的定性定量检测。由于样品基质的复杂性以及目标农药残留化合物通常为痕量甚至超痕量,样品前处理仍然是分析检测中不可避免的重要步骤,包括干扰基质的去除和目标化合物的分离、富集。样品前处理的效果直接影响到色谱、质谱检测的灵敏度和准确性。目前常用的样品前处理方法有液液萃取^[5]^、固相萃取^[6]^、QuEChERS^[7]^等,但液液萃取需要消耗大量的有机溶剂,固相萃取和QuEChERS对于目标化合物的选择性差,不能很好地去除杂质干扰。发展高选择性、易于制备、价格低廉的吸附剂是样品前处理的关键所在。

分子印迹聚合物(MIPs)是一种功能强大的选择性吸附剂,作为一种人工合成的仿生分子识别受体,其识别机理类似“锁钥模型”或者“抗原抗体作用”,因此也被称为“人造抗体”^[8]^。通过模板分子和功能单体预聚后,加入交联剂和引发剂或催化剂形成聚合物,去除模板之后形成的MIPs的孔洞与模板分子在空间形状、尺寸和功能基团互补^[9]^。MIPs对于给定的目标物质或者结构类似物具有预选择性,且制备简单,具有良好的热、化学、机械稳定性,价格低廉,具有良好的可重复性,因此广受人们的青睐,在手性识别^[10,11,12,13]^、生物化学传感^[14,15]^、催化降解^[16,17]^、药物传递^[18,19]^、分离纯化^[20,21,22]^等领域被广泛应用。

在极性农药残留的检测中,MIPs可以作为固相萃取(SPE)、固相微萃取(SPME)、磁性固相萃取(MSPE)、搅拌棒固相萃取(SBSE)等前处理方法的吸附剂,还可用于制备光、电、化学传感器、作为质谱检测的离子源基底和拉曼光谱的增强基底。本文主要介绍MIPs的制备策略及其在极性农药残留,包括新烟碱类、有机磷类、唑类、三嗪类、脲类等检测中的应用(见图1),并概述了MIPs在极性农药残留实际检测中所面临的挑战,对其未来发展做出了展望,希望为MIPs在极性农药残留检测中的研究提供一定参考。

**图1:**
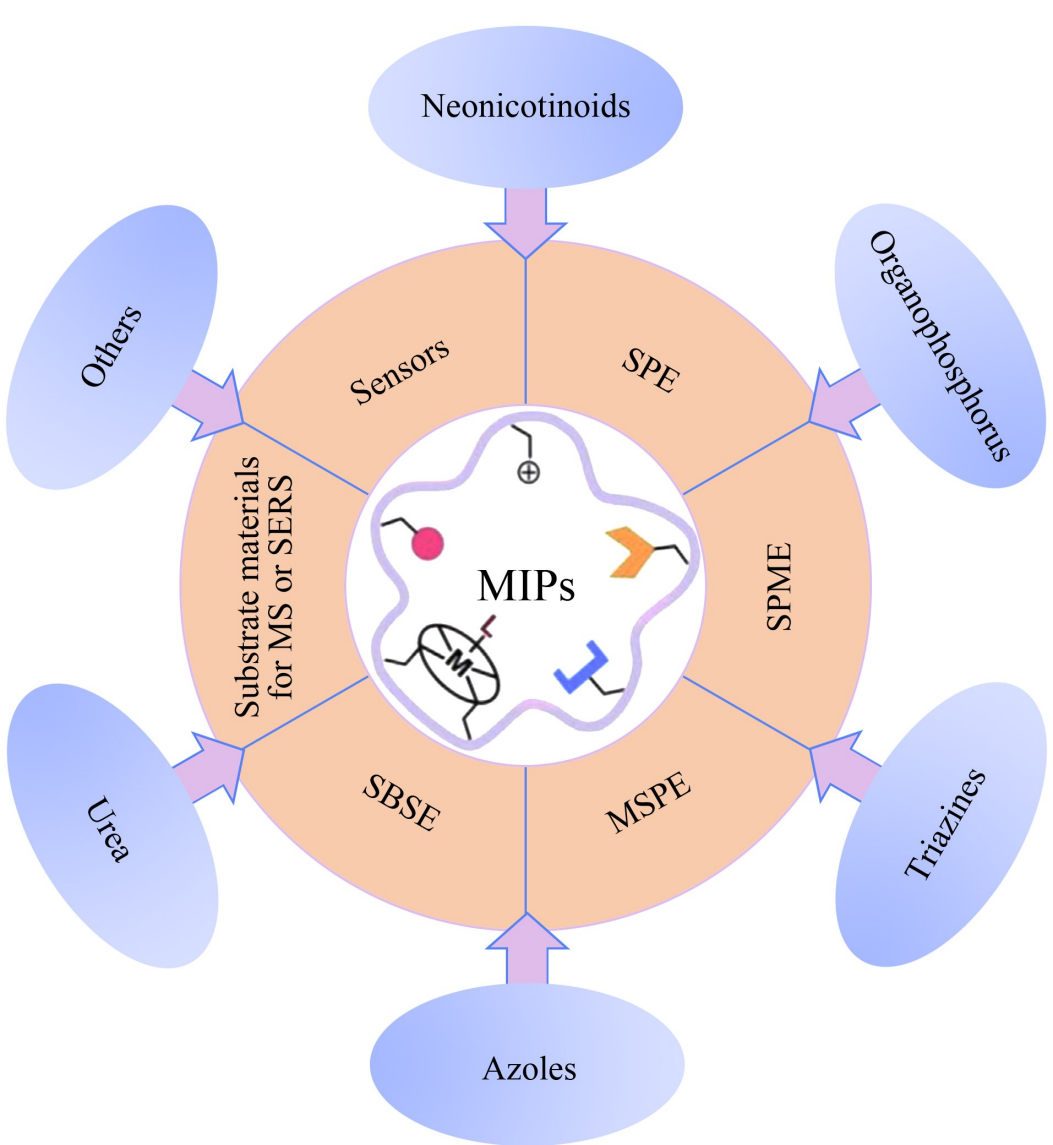
分子印迹聚合物在极性农药残留检测中的应用

## 1 分子印迹聚合物概述

分子印迹技术是Polyakov在1931年提出的^[23]^,在此基础上发展出了MIPs。分子印迹的方法通常有3种:共价印迹、半共价印迹和非共价印迹^[24,25]^(见图2)。前者是通过可逆的共价作用来稳定模板和单体形成的复合物,模板与单体之间具有很强的相互作用,因此比非共价方法制备的MIPs具有更加明确、更加均匀的吸附结合位点。在合成之后,通过在洗脱模板前断裂共价键,再从聚合物基质中去除模板形成对特定目标化合物有特异性和选择性的MIPs。共价印迹方法对合成条件,如温度和pH要求不太严格,但可逆的共价相互作用类型有限,且印迹过程通常比较繁琐,也不那么经济,因此限制了共价方法的使用。半共价印迹方法是在印迹过程中通过共价相互作用来稳定模板-单体复合物,而通过非共价相互作用重新结合目标化合物。非共价印迹是最常使用、最灵活的一种印迹方法,模板分子可以通过氢键、静电相互作用、疏水相互作用、*π-π*相互作用,偶极-偶极相互作用等多种相互作用与聚合物单体形成稳定的预聚混合物。与共价印迹方法相比,该方法需要大量的功能单体来促进较为稳定的模板-单体复合物的形成(模板和单体的摩尔配比通常为1:4),因此不可避免导致非选择性结合位点的密度更高。利用非共价印迹方法合成MIPs需要仔细优化实验条件,如模板、单体、交联剂的比例,致孔剂或溶剂的种类等,这与MIPs的选择性相关。

**图2:**
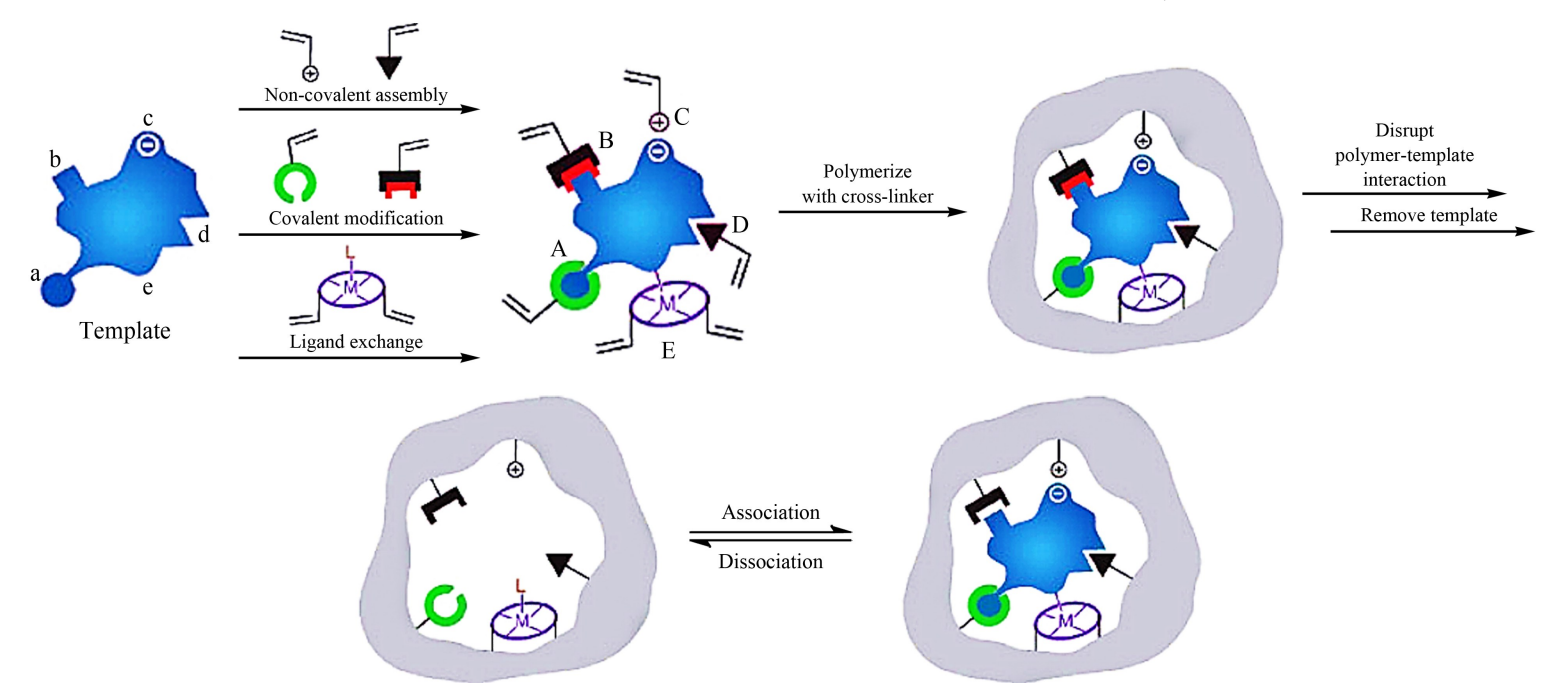
MIPs的制备过程和分子识别示意图^[9]^

### 1.1 分子印迹聚合物的传统制备策略

MIPs的制备一般需要3个步骤实现^[26]^: (1)在模板分子和功能单体之间形成预聚混合物;(2)在交联剂存在下,加入引发剂引发三维网状聚合物的形成;(3)除去模板,得到印迹聚合物。当模板被去除后,所获得的三维网状结构呈现出和模板互补的几何结构与官能团位点,从而能够特异性识别并选择性捕集目标化合物。

自由基聚合是制备MIPs使用频率最高的一种聚合方法^[25]^,其通常利用丙烯酸或者丙烯酸酯基单体,加入引发剂,在特定温度或者光照下即可启动聚合过程。基于自由基聚合机理,发展了本体聚合、悬浮聚合、沉淀聚合等聚合方法。利用本体聚合制备的MIPs使用前需要将合成的大块聚合物粉碎、研磨、过筛并在合适的溶剂中沉淀以除去更细的粉末颗粒,繁冗耗时的处理步骤可能对结合位点造成磨损,使特异性的空腔发生变形,且最终得到的颗粒粒度分布不均匀,限制了其在分析物提取中的应用。为了得到粒径均匀的聚合物颗粒,在MIPs的制备过程中引入了沉淀聚合和悬浮聚合。沉淀聚合是一种非均相聚合方法,增长的聚合物链不溶于其单体,从而以紧实的颗粒小球沉淀出来,制备出的聚合物粒径较为均匀,但需要大量致孔剂。在悬浮聚合过程中,聚合混合物的液滴悬浮在连续相(如水、矿物油等)中,单体液滴慢慢长大,逐渐成为聚合物固体颗粒。聚合过程温度控制较为容易,产物相对分子质量和颗粒大小分布相对均匀,但聚合过程比较复杂,必须使用分散剂,且聚合完成后难以从产物中完全去除。此外还有通过多步溶胀或者乳液聚合等方法制备MIPs。利用自由基聚合机理制备MIPs,过程简单高效,但聚合速率不受控制,且制备的MIPs结合位点分布不均匀。为了克服自由基聚合过程不可控的缺点,人们发展出了“受控/活性自由基聚合”(CRP)^[27]^,包括原子转移自由基聚合(ATRP)和可逆加成-断裂链转移聚合(RAFT)等,减缓了聚合速率,使得聚合过程可控。

### 1.2 分子印迹聚合物的新型制备策略

传统制备MIPs的方法操作简单,但得到的MIPs存在模板分子去除难、重复利用性差、印迹位点少、传质速度慢、难以与水环境相容等问题,近年来发展了许多MIPs的新型制备策略。传统MIPs的制备通常在有机溶剂中,大量的有机溶剂使用不仅危害环境,也会对实验人员造成潜在健康损害。利用溶胶凝胶法^[28]^,在使用纯水等绿色环保溶剂的环境友好条件下即可制备MIPs。传统本体聚合制备的MIPs极易将识别位点包裹在大块的聚合物内部,且不易将模板分子完全洗出;在使用过程中通常需要对其进行研磨,造成分子印迹空腔的变形和识别位点的损失。表面印迹策略^[29,30]^通过在固体支持物的表面形成印迹聚合物层,去除模板之后留下中空的分子孔道。识别位置位于载体表面,有利于目标化合物的进一步结合与洗脱,提高了MIPs的吸附效率,并保证了快速的传质动力学。此外,通过结合光敏单体^[31]^(如偶氮苯单体)和温敏单体^[32,33]^(常用*N*'-异丙基丙烯酰胺)等制备了环境敏感的智能MIPs,可以通过调节紫外/可见光或温度使得聚合物的结构或极性发生改变,实现待分析物的结合与释放开关,不仅避免了传统MIPs洗脱过程中有机溶剂和强酸强碱的使用,更加贴合环境友好的“绿色化学”倡导,还减少了对MIPs结构的破坏,从而提高了MIPs的使用寿命。

针对一些毒性大、价格昂贵的极性农药残留,也可采用假模版的策略,选取目标分子的结构类似物作为模板分子来制备相应的MIPs,实现待测物的选择性吸附和高灵敏检测^[33,34]^。在实际应用过程中,通常涉及一类农药残留的检测,若采用单一模板,则对模板分子以外的其他目标物交叉选择性小、提取效果差,因此使用双模板^[34]^甚至多模板分子^[35]^制备MIPs有助于一类结构类似物的选择性提取。此外,计算化学^[36,37,38]^也可作为制备MIPs的辅助手段。通过预先计算模拟目标化合物或者模板分子和反应单体与交联剂的相互作用,可以帮助筛选合适的单体与交联剂,优化模板、单体、交联剂的比例。

## 2 MIPs在极性农药残留检测中的应用

MIPs作为一种人工合成的高分子材料,具有与天然抗体相媲美的针对目标物的特异识别性和高效选择性。由于其易于制备、价廉、稳定性和重复性良好等优点,MIPs被广泛用于极性农药残留检测。除了用于样品前处理外,MIPs还可作为传感器的组成基元,与多种检测方法联用实现对极性农药残留的高灵敏检测。极性农药按照官能团不同可以分为新烟碱类、有机磷类、三嗪类、唑类、脲类等种类。针对不同种类极性农药的特性,发展了不同类型的MIPs和相应样品预处理技术和检测方法^[6,15,28,39-72]^(见图1和表1)。

**表1:** 分子印迹聚合物在极性农药残留检测中的应用

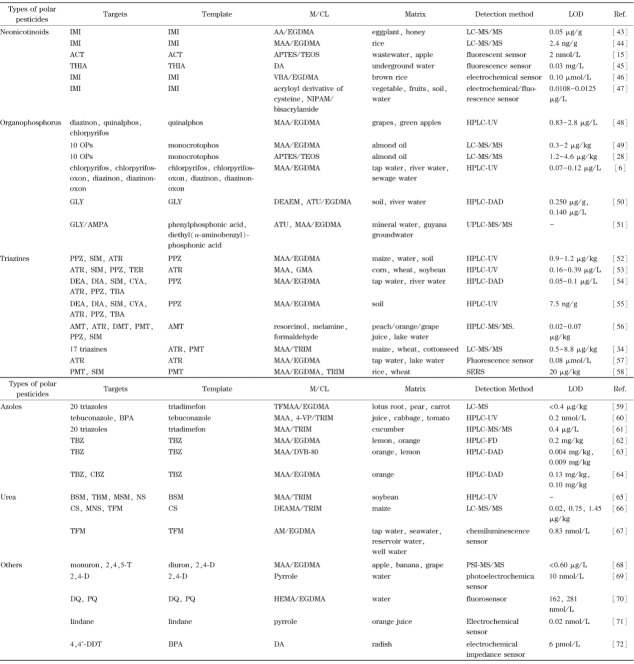

IMI: imidacloprid;ACT : acetamiprid; THIA: thiacloprid; OPs: or lyphosate;AMPA: aminomethylph cganophosphorus pesticides; GLY: g osphonic acid;PPZ: propazine; SIN M:simazine; ATR: atrazine; TER: terbutyn; DEA: desethylatrazine; DIA: desisop )ropylatrazine; CYA: cyanazine;TBA A: terbutylazine;AMT : ametryn; D MT : desmetryn; PMT : prometryn; BPA: bisphe- nol A;TBZ: thiabendazole; CBZ: carbendazim; BSM: bensulfuron-m nethyl; TBM : tribenuron-methyl;M SM:metsulfuron-methyl;NS:nic osulfuron ; Cs: chlorsulfuron;MNS monosulfu- ron;TFM: thifensulfuron-methyl;2,4,5-T: 2,4,5-trichlorophenoxyac etic acid; 2,4-D:2,4-dichlorophen oxyacetic acid; DQ: diquat; PQ: p henyltrichlo- araquat; 4,4'-DDT: 4,4'-dichlorodip roethane; M/ CL:monomer/ cross. ;linker;AA: acrylic acid; EGDMA: ethylene glycol dimethacrylate; MA A: methacrylic acid;APTES: 3-ami inopropyltriethoxysilane;TEOS: tetr raethyl ortho- silicate;DA: dopamine; VBA: p- vinylbenzoic acid; NIPAM: N-isopi ropyl acrylamide;DEAMA:2-( diet. hylamino ) ethyl methacrylate;ATU : N-allylthiourea; GMA: glycidyl m nethacrylate; TRM : trimethylolpropane trimeth: acrylate;TFMAA: trifluoromethyl a icrylic acid ; 4-VP: 4-vinylpyridine; DVB-80: divinylbenzene-80;AM : ac rylamide;HEMA: 2-hydroxyethyl m nethacrylate ; LC: liquid chromatography;MS: mass spectrometry ; GC: gas chrom atography;HPLC: high performanc e liquid chromatography ; UV: ultra violet; DAD: diode array detector; UPLC: ultra- high performance liquid chromato ography ; SERS: surface enhanced Ra man spectroscopy; FD: fluorescen ce detector;PSI: paper spray ioniz ation. -: not mentioned in the origir ial literature.

### 2.1 新烟碱类极性农药残留

新烟碱类农药作为一种系统性杀虫剂,由于其高效、广谱的杀虫活性以及对哺乳动物的低毒性,自20世纪90年代末以来成为在全球范围内最广泛使用的一类杀虫剂^[39,40]^。新烟碱类化合物被广泛用于多种农业生产作物,如蔬菜、柚子和核果、柑橘、水稻、棉花、玉米、马铃薯、甜菜、油菜和大豆等其他作物^[41]^。然而越来越多的研究报道了新烟碱类化合物的使用会对蜜蜂、鸟类、虾等非目标生物造成干扰,食用果蔬中新烟碱类农药残留对人类健康也存在潜在风险^[42]^。

基于MIPs对目标新烟碱类农药的特异性识别和选择性富集,已经发展了很多新型的MIPs用于新烟碱类农药的分离和检测。Kumar等^[43]^以功能化纳米Fe_3_O_4_粒子为磁芯,吡虫啉(IMI)为模板,制备了IMI磁性分子印迹聚合物(MMIP)吸附剂,用于蜂蜜和蔬菜样品中IMI的选择性分离。Chen等^[44]^将MIPs用作MSPE的吸附剂,建立了一种新的MIP-MSPE-LC-MS/MS方法,用于大米中IMI的选择性提取、分离和测定。与其他样品前处理方法相比,该方法简便、快速,重复性好,提取效率高,提取时间短,是一种很有发展前景的复杂样品前处理方法。

近年来,MIPs由于其对待测物的高选择性和耐酸耐碱、耐高温、适用于水相和有机相的高稳定性,在发展基于荧光/电化学传感器的目标物检测方面也广泛应用。与MIPs在固相萃取方法中使用颗粒形貌不同,MIPs作为传感器的传感元件,通常在碳量子点、电极、纳米材料、纤维等表面通过浸涂、纺涂、电聚合等方法制备成薄膜来使用。Poshteh Shirani等^[15]^以啶虫脒(ACT)为模板,通过溶胶凝胶法在高荧光特性的硅烷掺杂碳点(Si-CDs)表面制备了纳米复合荧光MIP材料(MIP@Si-CDs),用于废水和苹果中ACT的定量检测。MIP@Si-CDs的荧光信号在ACT浓度为7~107 nmol/L范围内表现出线性响应,检出限为2 nmol/L。Liu等^[45]^研制了一种基于测试条的噻虫啉定量检测传感器。在传感系统中,首先将氮掺杂石墨烯量子点(N-GQDs)浸入滤纸中。然后,多巴胺(DA)与噻虫啉在试纸条表面自聚合,形成均匀的聚多巴胺(PDA)膜。去除噻虫啉模板后,形成的PDA-MIP膜可特异性捕获并选择性识别噻虫啉,且捕获的噻虫啉能有效地提高N-GQDs的发光强度。Zhang等^[46]^以对乙烯基苯甲酸(VBA)为功能单体,在石墨烯(GN)表面诱导聚合均匀的MIP层,然后,将所得的MIP/GN分散液直接滴在抛光的玻碳电极(GCE)上,通过干燥,洗涤,电化学提取模板,制备了MIP/GN修饰的GCE,用于选择性和灵敏性电化学法检测糙米样品中IMI残留。该传感器的峰电流与IMI的浓度在0.5~15 μmol/L范围内呈线性关系,检出限为0.10 μmol/L。Kumar等^[47]^报道了一种用荧光染料(荧光素,FL)和印迹聚合物修饰的铕掺杂超顺磁性氧化铁纳米粒子(FL@SPIONs@MIP)高灵敏测定IMI的电化学/光学双传感器。所制备的传感器对IMI具有显著的选择性,已成功用于果汁、蔬菜、土壤、水等实际样品中IMI的检测。在传感器的制备过程中引入MIPs作为识别原件,结合了MIPs对目标新烟碱类农药的特异性识别能力,极大地降低了传统传感器在实际应用过程中的基质干扰,实现了对实际样品的快速、准确检测。

### 2.2 有机磷类极性农药残留

有机磷农药(OPs)是环境和食品污染的一个重要来源,因为它们具有广谱、高效、品种丰富和残留期短等特点而广泛用于对抗农业中大量的粮食作物昆虫。大多数有机磷农药是乙酰胆碱酯酶抑制剂,在被人体吸收时具有很高的急性毒性^[73]^。为了避免人类通过饮用水和食品接触有机磷农药残留,迫切需要一种有效的分离和检测有机磷农药残留的方法。在过去的几年里,人们基于分子印迹材料选择性高、吸附性强和可重复使用的特点,发展了许多从复杂基质中分离检测有机磷农药的方法。

Boulanouar等^[28,49]^以久效磷为模板,开发了多种能够选择性地从杏仁油中提取多种有机磷农药的分子印迹吸附剂。Sanagi等^[48]^以喹那磷为模板,非共价印迹聚合合成了MIPs,并将其作为固相萃取吸附剂,用于农业中广泛应用的3种OPs(二嗪农、喹那磷和毒死蜱)的HPLC检测。He等^[74]^在2015年首次以马拉硫磷为模板分子,甲基丙烯酸缩水甘油酯(GMA)为亲水性共单体,制备了一种新型的MIPs限制进入材料(RAM-MIPs)。将其作为固相萃取柱的吸附剂,对6种有机磷农药(马拉硫磷、乙氧磷、甲拌磷、特丁硫磷、乐果和非那米磷)的选择性与工业固相萃取柱相当甚至更高。Arias等^[6]^分别用毒死蜱、毒死蜱氧合物、二嗪农和二嗪农氧合物作为模板合成了4种MIPs,结果表明,以二嗪农为模板分子制备的MIPs对毒死蜱和二嗪农及其氧代衍生物具有最好的识别能力,能够选择性固相提取所有待测分析物。所开发的方法提供了令人满意的检出限(0.07~0.12 μg/L),且制备的MIPs显示出良好的可重复使用性(>50次重复使用)。将该方法应用于不同水样(自来水、污水、河水)中有机磷农药的萃取和富集,显示出良好的应用潜力。

草甘膦(GLY)是一种广谱性的极性有机磷农药,其性质稳定,不易光解和挥发,主要由土壤微生物氧化成为氨甲基膦酸(AMPA)和乙醛酸。GLY和AMPA因为极性高,不溶于大多数有机溶剂,且相对分子质量小,缺少发色团和荧光团,难以通过常规的光学技术进行检测^[50]^。发展高选择性的前处理材料预浓缩GLY和AMPA,并减少基质干扰对其检测有重要意义。Gomez-Caballero等^[50]^以烯丙基硫脲(ATU)和甲基丙烯酸2-二甲基氨基乙基酯(DMAEM)作为功能单体,未衍生化的GLY为模板,发展了一种具有良好水容性的磁性分子印迹搅拌棒,用于从水介质中选择性提取GLY。

### 2.3 三嗪类极性农药残留

三嗪类农药在农业上被广泛用于控制杂草生长,在土壤中半衰期长,甚至残留于地下水和地表水中,对动植物的危害很大,它们还可能通过生物链被人体吸收,对人体有潜在的“致畸、致癌、致突变”作用,可能导致皮疹、癌症、出生缺陷和内分泌紊乱等健康问题^[75,76]^,所以准确检测食品和环境中的三嗪类化合物显得尤为重要。

Geng等^[52]^以丙嗪(PPZ)为模板分子,制备了一种新型的纳米二氧化钛表面MIPs,结合实验表明MIPs对PPZ具有良好的吸附能力和较高的识别选择性,且对西马嗪(SIM)和阿特拉津(ATR)具有交叉选择性。Wang等^[53]^通过多步溶胀聚合,在中空分子印迹聚合物(H-MIPs)表面原位生长磁性Fe_3_O_4_纳米粒子,制备了新型的M-H-MIPs,并将其成功应用于玉米、小麦和大豆样品中4种三嗪类农药的萃取。Barahona等^[54]^制备了一种新型的分子印迹聚合物涂层中空纤维(MIP-HFs),结合分子印迹技术和液相微萃取技术分析水样中的三嗪类化合物。在实际应用过程中,将MIP-HFs直接浸入水样,搅拌一定时间,目标待测物先从样品中被液液萃取到甲苯中,然后扩散到MIP的特定结合位点。结合HPLC-DAD用于自来水和河水中七种三嗪类农药残留的检测,得到检出限为0.03~0.1 μg/mL,回收率在0.8%~6.9%。Diaz-Alvarez等^[55]^发展了一种简单的方法,制备用于SBSE的磁性分子印迹搅拌棒,结合HPLC-UV将其用于土壤中三嗪类农药残留的富集检测,回收率为2.4%~8.7%, 检出限低于7.5 ng/g。

为了实现多种类三嗪类农药的高效选择性富集,Wang等^[34]^以ATR和扑草净(PMT)为模板,制备了一种新型的双模板分子印迹聚合物(DMIPs),并将其作为一类特异性吸附剂应用于玉米、小麦和棉籽复杂样品基质中同时选择性固相萃取17种三嗪类除草剂及其代谢物,显示了DMIP-SPE在高通量提取和分析中的巨大潜力。Zhou等^[56]^以间苯二酚、三聚氰胺和甲醛为原料,采用一锅缩合法制备了磁性超亲水分子印迹树脂(MMIR),并将氨基功能化磁性纳米粒子引入到超亲水分子印迹树脂中,用于测定果汁和湖泊样品中的6种三嗪类化合物。

在三嗪类农药的检测中,除了被用于样品前处理的吸附剂外,MIPs还有多种应用。Nsibande等^[57]^耦合CdSeTe/ZnS量子点(QDs)和MIPs,开发了CdSeTe/ZnS@MIP荧光传感器,用于ATR的选择性检测。该传感器与ATR相互作用有较快的响应时间(5 min),荧光强度在2~20 mol/L的ATR的范围内线性猝灭,检出限为0.08 μmol/L,在水质监测中具有良好的应用前景。Yan等^[58]^以PMT为模板,设计了粒径均匀、吸附性能良好的MIPs,结合具有良好稳定性和增强性的金纳米颗粒表面增强拉曼光谱(Au-NPs-SERS),识别大米和小麦样品中的PMT和SIM。将MIPs用作SERS的基底,极大降低了在复杂样品检测中的基质干扰,得到了可靠的信号。该方法具有相当好的回收率(72.7%~90.9%), RSD为1.7%~7.8%,检出限为20 μg/kg。与非印迹聚合物相比,PMT的印迹因子为5.3, SIM的印迹因子为4.2。

### 2.4 唑类极性农药残留

唑类农药是一类高效的广谱类杀菌剂,主要包括咪唑类、三唑类和噻唑类。由于其高稳定性和较长的化学半衰期,唑类农药被广泛地应用于农业生产,以防治果蔬和粮食作物的各类真菌病,但其极易在粮食作物、土壤、环境水中残留积累^[77]^。有研究报道了唑类杀菌剂对水生生物和哺乳动物的生存、发育和生长、繁殖等行为造成干扰^[78,79]^;接触唑类杀菌剂及其残留物会对人类健康产生负面影响^[80]^。因此高效检测环境和食物中的唑类农药对保护生态环境和人类健康有重要意义。

Zhao等^[61]^以三唑酮为模板分子,甲基丙烯酸(MAA)为功能单体,三羟甲基丙烷三甲基丙烯酸酯(TRIM)为交联剂,乙腈(ACN)为成孔剂,采用沉淀聚合法制备了颗粒均匀的MIPs,将其作为SPE的吸附剂,同时测定黄瓜样品中20种三唑类杀菌剂和植物生长调节剂。Wu等^[60]^采用静电纺织技术将酸性双酚A(BPA)和碱性戊唑醇(TBA)同时封装在聚乙烯醇纳米纤维中,制备了具有多选择性的纳米纤维分子印迹膜(MIMs),用于同时提取蔬菜和果汁中电荷相反的痕量BPA和TBA。不同样品中两者回收率均高于70.33%, RSDs小于9.57%。

Martin-Esteban教授课题组^[62,63,64]^针对柑橘类水果中唑类农药的检测制备了多种MIPs。通过将印迹聚合混合物在乙烯基改性的二氧化硅磁性纳米粒子表面沉淀共聚制备了噻菌灵(TBZ)分子印迹核壳磁性纳米粒子,用于从柑橘样品中磁性固相萃取TBZ,并用高效液相色谱-荧光检测法(HPLC-FD)测定TBZ,其检出限为0.2 mg/kg^[62]^。通过将MIP微球填充到聚丙烯中空纤维段中,对柑橘样品中TBZ进行了SPME和净化。中空纤维膜能够保护MIP小球不受固体基质的影响,提取和净化目标物后不需要进一步过滤和/或离心步骤。橙子和柠檬中TBZ的检出限分别为0.004 mg/kg和0.009 mg/kg^[63]^。2019年,他们利用玻璃瓶塞作为模具,将磁性纳米粒子包埋在MIPs中,制备了一种用于SBSE的磁性搅拌棒,将其应用于橘子样品中TBZ和多菌灵(CBZ)的萃取,两种杀菌剂的检出限分别为0.13和0.10 mg/kg^[64]^。

### 2.5 脲类极性农药残留

脲类除草剂常作为土壤处理剂使用,易于被植物根部吸收,随蒸腾作用沿木质部上行传导至叶细胞,在光照下可能生成对植物有毒的物质,被认为是植物光合作用的典型抑制剂^[81]^。另外,脲类除草剂的水溶性都较高,极易残留于环境水体中。因此发展高效、灵敏的检测技术对环境和农产品样品中脲类除草剂残留的监测非常重要。

Tang等^[65]^以苄嘧磺隆(BSM)为模板制备了MIPs,建立了基于分子印迹固相萃取(MISPE)的前处理方法,用于选择性提取大豆样品中的BSM、苯磺隆(TBM)、甲磺隆(MSM)和烟嘧磺隆(NS)。She等^[66]^以氯磺隆(CS)为模板分子,甲基丙烯酸二乙氨基乙酯(DEAMA)为功能单体,TRIM为交联剂,采用沉淀聚合法合成了磺酰脲类MIPs,并建立了MISPE-HPLC-MS/MS法用于玉米样品中CS、单嘧磺隆(MNS)和噻吩磺隆(TFM)残留量的测定,回收率在75%~110%之间,检出限分别为0.02、0.75、1.45 μg/kg。

Xie等^[67]^以TFM为模板,采用沉淀聚合法制备了均匀的分子印迹微球,TFM通过氢键相互作用选择性地吸附在MIPs基体上,对鲁米诺-H_2_O_2_之间弱的化学发光(CL)反应有显著增强作用,基于此,建立了一种检测磺酰脲类除草剂TFM的新型MIP-CL传感器,该传感器具有动态范围宽,检出限低,选择性好,精密度高,可重复使用的优点,检出限为0.83 nmol/L。

### 2.6 其他极性农药残留

MIPs也被用于其他极性农药残留的检测。Chen等^[70]^设计了一种多功能荧光染料6,8-二羟基芘-1,3-二磺酸二钠(DHPDS),将其引入交联的聚(丙烯酸酯-丙烯酰胺)膜中,开发了基于MIPs的敌草快(DQ)和百草枯(PQ)选择性荧光传感器,该传感器可以通过电荷转移相互作用选择性检测水中的DQ和PQ除草剂,检出限分别为162和281 nmol/L。

2,4-二氯苯氧乙酸(2,4-D)是一种缺乏电化学活性的内分泌干扰物,直接的电化学技术难以检测。Shi等^[69]^采用聚吡咯MIPs作为识别元件,在TiO_2_纳米管上制备了具有优异光化学催化和分子识别能力的2,4-D光电化学传感器,用于多组分水样中2,4-D的选择性分析。

Pereira等^[68]^介绍了一种将MIPs和纸喷雾电离(PSI)相结合的新型MIP膜喷雾电离方法,该方法以灭草隆和2,4,5-三氯苯氧乙酸(2,4,5-T)为模板分子,在纤维素膜上直接合成MIPs,将其作为PSI-MS分析中电离的载体。该MIP膜可以特异性结合苹果、香蕉和葡萄等水果提取物中的敌草隆和2,4-D,避免了传统PSI在分析复杂样品时存在的灵敏度低、电离抑制等局限性。

## 3 结论与展望

作为一种人工合成的选择性材料,MIPs具有和天然抗体相当的识别亲和力,可以选择性分离捕集目标分析物,且其制备简单,造价低廉,可以重复性使用,因此在环境和食品等复杂基质中极性农药的分离分析中发挥着重要作用。MIPs可作为SPE、MSPE、SPME、SBSE等样品前处理方法的吸附剂,还可以作为质谱检测的离子源基质或者拉曼检测的信号增强基底。另外MIPs也经常被用于制备光、电、化学传感器,实现极性农药的快速检测。

目前MIPs在制备和实际的应用过程中,仍然存在模板分子的洗脱需要消耗大量有机溶剂,且难以完全去除,易造成模板泄露从而导致极性农药残留定性定量的不准确,以及重复利用率低,印迹位点少且不均匀,传质速度慢等缺点,急需要发展新的制备技术,设计新型的印迹复合材料来提高MIPs的吸附容量和对极性农药残留的识别效率。

未来MIPs在极性农药残留检测中的应用将发挥重要作用,基于MIPs的研究将集中在以下几个方面:(1)MIPs的合成方法的改善。将发展更加经济绿色的方式,如利用计算化学、化学计量学等在MIPs合成过程中辅助模板、功能单体、交联剂、温度、溶剂等影响因素的调控,以减少实验次数,并达到适合实际极性农药残留检测的MIPs的最佳条件。另外,通过引入可控活性自由基聚合等聚合方法以帮助制备更加均匀的MIPs微球也可改善其性能。(2)更多新型MIPs复合材料的设计,如亲水性MIPs的制备,以获得更好的水相容性,适用于水相复杂体系中极性农药的提取。设计对不同环境具有刺激响应功能的智能MIPs,通过光、热、pH、机械力等环境因素的改变,实现目标极性农药残留的可控吸附与释放,从而减少传统MIPs制备和应用过程中大量有机溶剂的使用,更加符合绿色化学发展的理念。(3)开发适用于宽范围极性农药检测的MIPs将是一个重要的研究方向。MIPs的良好通用性、易于制备、耗费低等优点为一些相对分子质量小、极性强的极性农药残留样品前处理和检测提供了更多可能性。
